# Predictors of Adherence to Smoking Cessation Medications among Current and Ex-Smokers in Australia: Findings from a National Cross-Sectional Survey

**DOI:** 10.3390/ijerph182212225

**Published:** 2021-11-21

**Authors:** Amanual Getnet Mersha, Michelle Kennedy, Parivash Eftekhari, Gillian Sandra Gould

**Affiliations:** 1 School of Medicine and Public Health, The University of Newcastle, University Drive, Callaghan, NSW 2308, Australia; michelle.bovill@newcastle.edu.au (M.K.); parivash.eftekhari@newcastle.edu.au (P.E.); 2 Hunter Medical Research Institute, Lot 1, Kookaburra Circuit, New Lambton Heights, NSW 2305, Australia; 3 Faculty of Health, Southern Cross University, Coffs Harbour, NSW 2450, Australia; gillian.gould@scu.edu.au

**Keywords:** adherence, cigarette, quitting, smoking, smoking cessation medications

## Abstract

Background: Adherence to smoking cessation medications (SCMs) improve the rate of successful quitting. This study aimed to evaluate the level of adherence to SCMs and associated factors among smokers and ex-smokers in Australia. Method: A cross-sectional study using an online survey was conducted in Australia. Descriptive statistics were used to present the overall characteristics of participants. Cross-tabulation with Pearson’s chi-square test was performed to evaluate the possible associations between factors. To explore barriers and facilitators of adherence to SCMs, logistic regressions were conducted. Results: Among 201 participants, 57 (28.4%) were found to be adherent to SCMs. The odds of being adherent were found to be higher among participants with good social support (AOR = 3.28, 95% CI of 2.30–6.27). Participants who did not experience anxiety symptoms had higher odds of being adherent to SCMs as compared to smokers who had anxiety symptoms (AOR = 4.41, 95% CI of 3.64–14.68). Having previous experience of using SCMs improved adherence four-fold (AOR = 3.87, 95% CI of 1.11–13.44). Level of nicotine dependence showed a direct association with adherence (AOR = 3.53, 95% CI of 1.40–8.95). Not relapsing while on the medications improved adherence (AOR = 2.88, 95% CI of 1.21–6.88). Conclusion: In a study of smokers and ex-smokers in Australia, the self-reported level of adherence to SCMs was found to be low. Adherence was associated with social, psychological, and medication-related factors. Smoking cessation interventions are recommended to include strategies that can address medication adherence.

## 1. Background

During the last few decades, smoking cessation programs and interventions have become an urgent need due to the global increase in health hazards from smoking [1]. Pharmacological interventions for tobacco dependence are both safe and effective [2]. Clinical guidelines recommend the use of smoking cessation medications such as nicotine replacement therapies (NRT), varenicline, or bupropion [3,4]. Participants using smoking cessation medications have been found to be 1.5 to 2 times more likely to quit smoking than those using behavioral therapy alone [5].

The rate of successful smoking cessation is higher among individuals who have consistent and adherent smoking cessation medication use [6]. A meta-analysis was conducted and published by the authors of this study in 2021 to evaluate the effect of adherence on the success of smoking cessation. The study indicated that adherence to NRT doubles the rate of successful smoking cessation [7]. The biochemically verified rate of smoking cessation was higher among individuals who were adherent to bupropion therapy as compared to non-adherent counterparts [8]. Similarly, adherence to varenicline was associated with a twofold increase in quit rates [9].

A systematic review incorporating studies conducted mainly in the USA and UK demonstrated the importance of multiple personal, social, and environmental factors [10]. The level of nicotine dependence, withdrawal symptoms, perceptions about medications and quitting, alcohol use, stress, depression, and social support were found to be associated with adherence to smoking cessation medications [10]. Smokers’ beliefs about nicotine and medications, adverse effects of the medicine, and attending more counseling sessions were associated with adherence to NRT and bupropion [11]. There are conflicting findings regarding the association between the intensity of nicotine dependence and adherence to smoking cessation medications. Some studies found better adherence among individuals with a higher level of nicotine dependence [12,13,14,15], whereas other studies reported that higher dependence reduced the rate of adherence to smoking cessation medications [16,17,18].

In Australia, there has been a significant downward trend of smoking prevalence from 24% in 1991 to 12.2% in 2016 [19]. Absolute reductions in smoking rates have largely been due to a decrease in smoking initiation. For example, the proportion of young adults aged 18–24 who never smoked increased from 58% to 80% between 2001 and 2019 [19]. The rate of ex-smokers improved only by 2% between 1991 and 2019 [19]. This could be partly explained by the under-utilization of smoking cessation supports including medications as well as a reduction in uptake of smoking due to tobacco control policies. However, relatively few comprehensive approaches are being implemented in Australia within smoking cessation services compared with other countries [20].

To the best of our knowledge, the level of adherence and factors associated with adherence to smoking cessation medications have not been investigated in Australia. Understanding the barriers and facilitators of adherence to smoking cessation medications is crucial for the development of comprehensive and effective interventions that can improve the success of smoking cessation.

## 2. Methods

A national cross-sectional study was conducted to assess adherence to smoking cessation medication use and its associated factors among adult smokers and recent ex-smokers in Australia from January 2021 to July 2021.

### 2.1. Sampling and Recruitment

The sample size was calculated using the single population proportion formula with the assumption of a confidence level of 95% and a margin of error of 5%, and the prevalence of smoking in Australia was taken as 11.6% in 2019 [21]. Thus, a minimum sample of 158 people would be required to detect a true association or effect between participants’ characteristics and adherence to smoking cessation medications.

A 61-item online survey was administered through a consumer panel provider, Pureprofile Pty Ltd (https://www.pureprofile.com/ (accessed on 16 April 2021). Pureprofile was contracted to distribute the survey invitation. Individuals who were registered to the survey provider and reported smoking were contacted and provided with the screening questions. Eligible participants were then provided with the survey link. The online survey was designed and disseminated using Qualtrics software (Qualtrics, Provo, UT, USA). Adults (aged 18 years old and above) who were current smokers or recent ex-smokers and quit smoking in the last 12 months by using smoking cessation medications (NRT, varenicline, bupropion) during their most recent quit attempt were invited for participation. Participants were reimbursed with 15 AUD in cash for their time through the online survey provider, Pureprofile.

### 2.2. Measures

Participants’ socio-demographic data such as age in years, gender (male or female), geographic remoteness, region, marital status, level of education, and other characteristics were gathered. The age of the participants was collected as a continuous variable and categorized into two classes: as young and middle-aged adults (18–55 years old) and older adults (>55 years old). A previous study using this age group classification illustrated a significant difference in adherence between the age groups [22]. The Australian Statistical Geography Standard-Remoteness Area guideline was used to classify geographical remoteness by using participants’ postal codes [23]. The 2016 socioeconomic index for areas (SEIFA) created by the Australian Bureau of Statistics (ABS) to evaluate the relative socioeconomic disadvantage (IRSD) was determined using postal codes [24]. At an Australian national standard of 1000, a score of lower than 1000 indicates relatively greater socioeconomic disadvantage, and a score of >1000 was categorized as “nil/no” socioeconomic disadvantage [25]. The educational level was split into four categories: never attended formal education, completed primary education, completed secondary education, and completed college and above. Marital status was collected as married, never married or separated/divorced/widowed. Geographical remoteness was measured in five classes: major cities, inner regional, outer regional, remote, and very remote in Australia.

The level of nicotine dependence was assessed using the Fagerstrom Test for Nicotine Dependence (FTND). A high level of nicotine dependence is defined as a score of 6 and above out of a maximum score of 10 [26]. Social support during the quit attempt was evaluated using the Oslo social support scale (OSSS-3). The scale assesses the structural and functional level of social support using 3 questions with a possible score of 3 to 14. A score of 3 to 8, 9 to 11, and 12 to 14 indicates poor, moderate, and strong social support, respectively [27].

The type of medications used to assist smoking cessation were evaluated and reported. Medications could be prescribed by health care providers or bought over the counter. In Australia, most NRTs can be purchased over the counter; however, bupropion and varenicline are prescription-only medications listed in the pharmaceutical benefits scheme so individuals can obtain them at a subsidized rate.

Self-reported psychological symptoms were assessed using the depression, anxiety, and stress scale (DASS-21) [28]. This instrument was evaluated among individuals with substance use disorder and found to be sensitive and specific [29]. Each of the three DASS-21 scales contains seven items, divided into subscales with similar content. Scores for depression, anxiety, and stress are calculated by summing the scores for the relevant items. For each question, participants selected 0, 1, 2, or 3 if the stated symptom did not happen at all, happened some of the time, happened a considerable amount of the time, or most of the time, respectively [28].

To assess stress, a score of less than 11, 11 to 18, 19 to 26, 27 to 34, and more than 35 represents none, mild, moderate, severe, and extremely severe symptoms of stress, respectively. For anxiety, a score of less than 7, 7 to 9, 10 to 14, 15 to 19, and more than 20 indicates the presence of no, mild, moderate, severe, and extremely severe symptoms of anxiety, respectively. A score of less than 10, 10 to 12, between 13 to 20, 21 to 27, and more than 28 out of a maximum score of 42 represent no, mild, moderate, severe, and extremely severe depression, respectively [28].

### 2.3. Outcome Variables

There are no standard definition and assessment measures to evaluate adherence to smoking cessation medications to date [30]. Hence, previous studies used different definitions and measures such as daily use, pill count, and duration of medication use to measure adherence. the following definition was therefore used in this study to evaluate adherence.

Adherence was assessed using the self-reported duration and weekly consumption pattern of smoking cessation medications during participants’ most recent quit attempts. Participants were labeled as adherent if they used smoking cessation medications for at least 4 weeks and on average for 5 or more days per week. Combining duration and weekly usage patterns gives better information than evaluating the duration of treatment alone [31]. Moreover, previous studies have also used this operational definition to define adherence [22,32].

**Figure 1:**
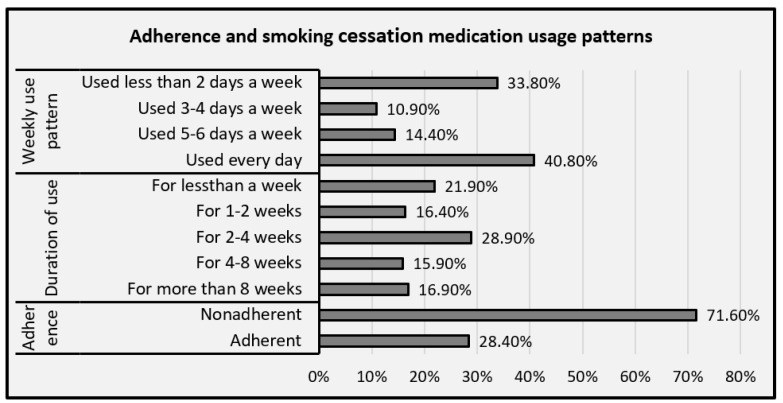
Adherence and smoking cessation medication usage patterns.

To provide a more detailed and informative report, we included a figure to illustrate adherence based on the number of days and duration of medication use measured in weeks (Figure 1).

Nonadherence was defined as medication use for fewer than 4 weeks or fewer than 5 days a week. If a participant used the medication for 4 weeks but fewer than 5 days a week on average, they were categorized as nonadherent. Likewise, if a participant used the medication for 5 days or more per week but for a total duration of less than 4 weeks, they were labeled nonadherent.

### 2.4. Data Analysis

Data were entered into and analyzed using Stata software (V16, Stata Corp LP, College Station, TX, USA). Descriptive statistics using frequency and percentages were used to present the overall characteristics of participants. Cross-tabulation with Pearson’s chi-square test was used to evaluate the possible association of various factors and adherence to smoking cessation medications.

Factors associated with adherence to smoking cessation medications were determined using logistic regression. Univariate analyses were conducted to evaluate the unadjusted effect of factors on adherence. Factors with a significant association from the univariate analyses were further included in the multivariable forward stepwise logistic regression. To declare a significant association, a *p*-value of 0.05 was used as a cut-off point, and the results were expressed by using an odds ratio (OR) with a 95% confidence interval (CI).

### 2.5. Ethics Approval

This study was approved by the University of Newcastle Human Research Ethics Committee, approval Number H-2021-0073. Participants were provided with an information sheet to inform them of the study objective and their rights. Participants were informed that participation was entirely voluntary. Volunteer participants signed an implied consent to start the survey. In case participants required emotional support after being exposed to the survey, contact details and links to some available free-of-charge services were provided to them in the information sheet and at the end of the survey.

## 3. Results

### 3.1. Socio-Demographic Characteristics of Participants

A total of 201 participants who used smoking cessation medications in the last 12 months for the purpose of quitting completed the survey and were included in the study. The median age of participants was 47 years. The majority of the participants were female (70.6%). Nearly half of the participants (47.8%) were from areas with greater socioeconomic disadvantage—a score of <1000, based on the Australian Bureau of Statistics (ABS) relative socioeconomic disadvantage (IRSD) score.

Half of the participants (53.2%) reported having good social support during their quit attempt; i.e., they scored 9 and above on the Oslo Social Support scale. A total of 63.2% participants reported drinking alcohol during their recent quit attempt; of these, 17.9% reported drinking alcohol-containing beverages at least 4 days a week and 27.4% reported drinking alcohol 2 to 3 times per week. The chi-square test illustrated a possible association between adherence to smoking cessation medications and various participant characteristics such as age, employment status, remoteness, alcohol use, social support, and psychological symptoms (Table 1).

### 3.2. Psychosocial Characteristics of Participants

The most frequently reported psychological symptoms experienced during a participants’ quit attempt were anxiety and stress, at 43.8% and 40.5%, respectively. Among the participants who had experienced anxiety, 13.4% and 11.9% participants experienced moderate and severe anxiety symptoms, respectively. Among the 81 participants who had experienced stress, 6% reported a moderate level of stress during their quit attempt. Moreover, 36.3% of participants reported depressive symptoms, and one in five of the participants (22.9%) who reported depressive symptoms experienced at least a moderate level of symptoms (Table 1).

To evaluate the association of the abovementioned psychological symptoms on medication adherence, further categorization into a binary classification was conducted for each of the three disorders. The classification was into groups of those participants with any symptoms and those who did not experience psychological symptoms.

### 3.3. Adherence to and Pattern of Smoking Cessation Medications Use

Among the participants, 16.9% reported they used the medications for at least 8 weeks; 15.9% used them for 4 to 8 weeks; 28.9% used them for 2 to 4 weeks; 16.4% used them for 1 to 2 weeks; and the remaining 21.9% participants used the medication for less than 1 week. The average weekly usage patterns of smoking cessation medications showed that 40.8% of participants used the medications every day of the week; 14.4% used them for 5 to 6 days a week; 10.9% used the for 3 to 4 days per week; and 33.8% of participants used them for 2 or less days per week. As per our definition of adherence (usage of smoking cessation medications for a duration of at least 4 weeks with weekly usage for at least 5 days a week), 28.4% of participants were found to be adherent to smoking cessation medications (Figure 1).

### 3.4. Smoking, Quit Attempt, and Beliefs about Smoking Cessation Medications 

Among the participants, 134 (66.6%) were current smokers and 67 (33.3%) were ex-smokers. Around 39% of the participants had a moderate to high level of nicotine dependence at the start of their quit attempt; i.e., a score of 5 and above on the Fagerstrom Test for Nicotine Dependence (FTND). The majority of participants (85.6%) had at least one previous quit attempt, of which 65.2% had used smoking cessation medications during their previous quit attempts. The vast majority (71.6%) of participants used NRT in their recent quit attempt, whereas varenicline and bupropion were used by 19.9% and 8.5% of participants, respectively. Around 72.6% and 54.7% of participants believed smoking cessation medications were safe and effective, respectively. Relapse during a quit attempt characterized by smoking for more than two days per month while on the smoking cessation medications was reported by 64.2% of the participants. Among the participants, 82% reported craving for a cigarette during their quit attempt. The presence of other smokers at home during their quit attempt was reported by 41.3% of the participants. The chi-square test showed a possible association between adherence to smoking cessation medications and the extent of nicotine dependence, previous use and type of smoking cessation medication, forgetfulness, and relapse of smoking (Table 2).

### 3.5. Factors Associated with Adherence to Smoking Cessation Medications

Thirteen factors from the univariate analyses showed significant associations with self-reported adherence to smoking cessation medications. The variables were further adjusted using multivariable analysis, as illustrated in Table 3. Five factors showed a significant association with adherence in the multivariable analysis.

The odds of being adherent to SCMs were reported to be higher among participants who reported good social support during their quit attempt (AOR = 3.28, 95% CI of 2.30–6.27, *p*-value 0.012). Although all the assessed psychological variables showed an association in the univariate analysis, only anxiety showed a significant association after adjustment. Participants who did not experience any anxiety symptoms had improved adherence by odds of more than four times compared to participants who had experienced anxiety symptoms (AOR = 4.41, 95% CI of 3.64–14.68, *p*-value 0.016).

Having experience of using smoking cessation medications in previous quit attempts improved adherence nearly four-fold (AOR = 3.87, 95% CI of 1.11–13.44, *p*-value 0.033). Furthermore, participants with moderate to high nicotine dependence were more adherent than those with low nicotine dependence (AOR = 3.53, 95% CI of 1.40–8.95, *p*-value 0.008). Relapse was negatively associated with medication adherence. Participants who did not relapse had improved adherence compared to those who reported relapse, characterized by smoking more than two days a month during their quit attempt (AOR = 2.88, 95% CI of 1.21–6.88, *p*-value 0.017) (Table 3).

## 4. Discussion

Overall, this study showed a low level of adherence to smoking cessation medications among smokers and ex-smokers. Adherence to smoking cessation medications was associated with the level of social support, the extent of nicotine dependence, anxiety, relapse, and experience of previous use of the smoking cessation medications. The finding from this study agrees with a systematic review conducted and published by the authors that indicated the importance of multiple social, psychological, and medication-related factors in regulating adherence to smoking cessation medication [10].

Three in 10 (28.4%) smokers and ex-smokers in Australia were found to be adherent to smoking cessation medications. In a 2021 meta-analysis that included studies conducted mainly in the USA and UK, 26% of participants were found to be adherent to NRT. The meta-analysis showed higher rates of quitting among participants who were adherent to the medications and recommended that smoking cessation programs should consider adherence as one of the core components of the intervention [7]. In a study conducted among Korean smokers, 28.2% of participants used Varenicline for the recommended duration [33]. Adherence was reported to be higher in randomized controlled trial participants, at 61% and 74% among users of NRT [7] and bupropion [8], respectively. RCT participants are likely to be more motivated to quit and obtain the necessary medication-related information, which may have contributed to this disagreement [7]. This could be due to various factors of smokers and health care providers, such as lack of time, lack of training, and motivation [34]. A study conducted in the UK showed disparities between the expressed needs of smokers and the health care providers’ beliefs regarding the support required for adherence to NRT [35]. There is also a substantial discrepancy in asking about smoking status and assisting smokers to quit which may have affected the rate of adherence to the medications [34,36]. Smoker-related factors associated with adherence to smoking cessation medications are discussed in the following paragraphs.

Experience of using smoking cessation medications during previous quit attempts was associated with higher rates of adherence. Participants who reported previous use of the medications were more likely to have a better motivation to quit and use the SCMs, which may have confounded this finding. Our finding is in line with findings from reviews [10,11] and original studies conducted in China [22] and the UK [37]. Having previous exposure to smoking cessation medications improves adherence by helping participants to establish a realistic expectation about the effectiveness of the medications. It also helps individuals to select the type of medication they are comfortable with and prepare themselves for possible medication side effects such as headache, skin irritation, insomnia, and others [11,12,38]. Lack of knowledge about the side effects and unrealistic expectations was associated with poor adherence [39].

A higher level of nicotine dependence is positively associated with adherence to smoking cessation medications. Mixed results have been reported in the literature regarding the relationship between nicotine dependence and adherence. The finding in this study is in line with reports from randomized controlled trials [14] and population-based studies [12,13,15] conducted in the USA that reported higher adherence among smokers with higher levels of nicotine dependence. This could be due to a difference in the perceived need for smoking support medications. On the contrary, other studies reported that higher dependence reduced the rate of adherence to smoking cessation medications [16,17,18].

Relapsing to smoking reduced the rate of adherence to smoking cessation medications. The association between relapse and adherence can be bidirectional; i.e., relapse can be both a cause and effect of non-adherence. The demonstrated effect of relapse on adherence in this study may have been influenced by reverse causality. Hence, this should be taken into account during interpretation of this finding. Studies that reported an inverse association between nicotine dependence and adherence reported lower rates of quitting among individuals with more nicotine dependence and argue smoking status has a mediating effect between adherence and nicotine dependence [17,40]. This may be due to fear of compound toxicity from the medication and the smoke [16]. Participants may also start to doubt themselves, and this may affect their self-efficacy, which could reduce their motivation to use the medication and quit smoking [41]. Continuing the medications promotes recovery from relapse and the attainment of abstinence [40].

A greater social support network is associated with better adherence to smoking cessation medications. A systematic review aimed at assessing factors associated with adherence to NRT indicated a positive impact of having social support during a quit attempt [10]. Findings from patients with chronic medical conditions such as diabetes [42] and heart disease [43] demonstrated that both structural and functional components of social support are vital to medication adherence. Social support may improve medication adherence directly, by having someone to remind the individual to take the medications appropriately, and indirectly, by motivating them to stay smoke-free [44] and improve their self-efficacy [45].

The relatively higher levels of psychological symptoms can be partially explained by the proportion of the male to female ratio in the data and the overlapping of the study with the COVID pandemic [46]. The 2020 Australian Institute of Health and Wellbeing (AIHW) report indicated that a higher proportion of females than males (22% compared with 18%) had experienced symptoms in the previous 12 month period [47]. Symptoms of depression during quit attempts were reported to be as high as 40% in other studies [48]. Moreover, this study included participants who had experienced a quit attempt in the previous 12 months, in which time the COVID pandemic imposed a significant mental health challenge [49]. The experience of anxiety symptoms during a quit attempt negatively affected the rate of adherence to smoking cessation medications. Participants with no symptoms of anxiety had a four-fold increase in the odds of adherence. Similarly, other studies have also indicated the detrimental effect of psychological symptoms during quit attempts on adherence to medications [10,11,50]. This effect could be mediated by a higher level of relapse among participants with mental illnesses as compared to individuals with no psychological symptoms (24% vs. 12%) [51,52]. Integrated interventions directed at smoking cessation and improving mental health have demonstrated an improved adherence to treatment and quitting [53].

## 5. Strength and Limitations of the Study

Although studies have indicated that adherence improves successful smoking cessation, there has been no study conducted to explore the factors associated with adherence in Australia until now. This study is the first to explore the barriers and facilitators of adherence to smoking cessation medications in Australia. It will provide a framework for further large-scale clinical trials and population-based studies.

This study has several limitations that should be noted. Most of the participants were urban residents, which could be due to differences in internet access. Participants of online surveys are more likely to be active users of technology and have exposure to smoking and medication-related information.

As the survey was an online survey and potential participants could open and close the survey link at any time, it is difficult to determine the exact response rate. However, the sociodemographic characteristics of participants in this study are comparable to the national data. In the 2019 report of the Australian Institute of Health and Welfare, the proportion of daily smokers was higher in middle-aged adults than older adults (18.4% in the age group between 40–49 years as compared to 11.9% among adults from 60–69 years old). The same data reported that around 31% of smokers completed college-level education and above [54]. According to the Australian national census, New South Wales (32%) and Victoria (24%) are the most populated regions in Australia [55]. Despite these limitations, this study illustrated essential factors affecting adherence to smoking cessation medications and provided new information in Australia.

The findings may have been influenced by limitations inherent to a cross-sectional study design, such as establishing a temporal relationship and recall bias, which may have affected reported adherence rates and associated factors. As this is a cross-sectional study, relapse may have a reverse causality effect on adherence; i.e., relapse can be a cause or effect of non-adherence. Future studies are recommended to control for reverse causality by including participants who did not relapse or by performing the analysis to the point of relapse. Additionally, the issue of polypharmacy in individuals with chronic medical conditions was not investigated, which may have been impacted adherence.

## 6. Conclusions and Recommendations

In conclusion, we found a low level of adherence to smoking cessation medications among smokers and ex-smokers in Australia. Medication adherence was associated with social, psychological, and medication-related factors, as well as the extent of nicotine dependence. Knowledge of how medications are used for smoking cessation is essential to guide further interventions. A further study with larger national representative data is recommended to achieve stronger evidence.

## Figures and Tables

**Table 1:** Psycho-social and demographic characteristics of participants (*n* = 201).

Variables	Adherence *n* = 201		*p*-Value
	No Frequency (%)	Yes Frequency (%)	
Age	Median—47 years old		0.002 *
18–55	100 (69.4%)	26 (45.6%)	
≥56	44 (30.6%)	31 (54.4%)	
Gender			0.552
Female	100 (69.4%)	42 (73.7%)	
Male	44 (30.6%)	15 (26.3%)	
Education level			
Completed primary or secondary school	71 (49.3%)	28 (49.1%)	0.981
Completed college and above	73 (50.7%)	29 (50.9%)	
Employment status			0.037 *
Employed	89 (61.8%)	26 (45.6%)	
Unemployed	55 (38.2%)	31 (54.4%)	
Marital status			0.066
Married	60 (41.7%)	22 (38.7%)	
Never married	48 (33.3%)	12 (21%)	
Divorced/Widowed/Separated	36 (25%)	23 (40.3%)	
Remoteness			0.005 *
Major cities of Australia	116 (80.6%)	35 (61.4%)	
Regional and remote Australia	28 (19.4%)	22 (38.6%)	
Socioeconomic disadvantage (SEIFA)			0.808
High	76 (52.8%)	29 (50.9%)	
Low	68 (47.2%)	28 (49.1)	
State of residence			0.088
New South Wales	59 (50%)	10 (17.5%)	
Victoria	35 (24.3%)	17 (29.8%)	
Queensland	19 (13.2%)	15 (26.3%)	
South Australia	14 (9.7%)	8 (14%)	
Western Australia	17 (11.81%)	7 (12.3%)	
Drinking alcohol			0.015 *
No	59 (41%)	15 (26.3%)	
Yes	85 (59%)	42 (73.7%)	
Social support			
Good social support (OSSS-3 score ≥ 9)	55 (38.2%)	43 (75.4%)	0.001 *
Poor social support (OSSS-3 score ≤ 8)	89 (61.8%)	14 (24.6%)	
Stress			0.004 *
No stress (DASS score ≤ 18)	76 (53.1%)	43 (75.4%)	
Had symptoms of stress(DASS score ≥ 19)	67 (46.9%)	14 (24.6%)	
Anxiety			0.001 *
No anxiety (DASS score ≤ 9)	64 (44.4%)	49 (86%)	
Had symptoms of anxiety (DASS score ≥ 10)	80 (55.6%)	8 (14%)	
Depression			0.001 *
No depression (DASS score ≤ 12)	79 (54.9%)	49 (86%)	
Had symptoms of depression (DASS score ≥ 13)	65 (45.1%)	8 (14%)	

DASS—Depression, Anxiety, and Stress Scale; OSSS—Oslo Social Support Scale; *p*-value—chi square test; *—significant *p*-value.

**Table 2:** Smoking characteristics, quit attempts, and beliefs about SCMs among smokers and ex-smokers in Australia (*n* = 201).

Variables	Adherence *n* = 201		*p*-Value
	No Frequency (%)	Yes Frequency (%)	
Level of nicotine dependence			0.002 *
Low dependence (FTND score ≤ 4)	98 (68.1%)	25 (43.9%)	
Moderate to high dependence (FTND score ≥ 5)	46 (31.9%)	32 (56.1%)	
Ever had quit attempt previously			0.060
Yes	119 (82.6%)	53 (93%)	
No	25 (17.6%)	4 (7%)	
Ever used SCMs during a previous quit attempt			0.003 *
Yes	83 (69.7%)	48 (90.6%)	
No	36 (30.3)	5 (9.4%)	
Type of SCM in the current quit attempt			0.027 *
NRT	108 (75%)	36 (63.2)	
Varenicline	22 (15.3%)	18 (31.5%)	
Bupropion	14 (9.7%)	3 (5.3%)	
Ever forget to take the SCMs			0.016 *
Yes	75 (52.1%)	19 (33.3%)	
No	69 (47.9%)	38 (66.7%)	
Believe SCMs are safe			0.107
Yes	100 (69.4%)	46 (80.7%)	
No	44 (30.6%)	11 (19.3%)	
Believe SCMs are effective			0.570
Yes	77 (53.5%)	33 (57.9%)	
No	67 (46.5%)	24 (42.11%)	
Intentionally missed SCMs			0.026 *
Yes	65 (45.1%)	16 (28.1)	
No	79 (54.9%)	41 (71.9%)	
Skip SCMs when feeling better			0.063
Yes	84 (58.3%)	25 (43.9%)	
No	60 (41.7%)	32 (56.1%)	
Relaps			0.043 *
Yes	99 (68.7%)	30 (52.6%)	
No	45 (31.3%)	27 (47.4%)	
Experienced sleeping problems since quitting			0.420
Yes	62 (43.1%)	21 (36.8%)	
No	82 (56.9%)	36 (63.2%)	
Experienced eating problems since quitting			0.640
Yes	68 (47.2%)	29 (50.9%)	
No	76 (52.8%)	28 (49.1%)	
Do physical exercise during the quit attempt			0.140
Yes	67 (46.5%)	20 (35.1%)	
No	77 (53.5%)	37 (64.9%)	
Experienced craving since quitting			0.086
Yes	114 (79.2%)	51 (89.5%)	
No	30 (20.8%)	6 (10.5%)	
Others smoking inside living home			0.625
Yes	61 (42.4%)	22 (38.6%)	
No	83 (57.6%)	35 (61.4%)	

FTND—Fagerstrom Test for Nicotine Dependence; SCMs—smoking cessation medications; NRT—nicotine replacement therapy; *p*-value—chi-square test; *—Significant *p*-value.

**Table 3:** Factors associated with self-reported adherence to SCMs among smokers and ex-smokers in Australia aged 18 and above (*n* = 201).

Variables	Adherence *n* = 201		COR (95% CI)	AOR (95% CI)
	No Frequency (%)	Yes Frequency (%)		
Age				
18–55	100 (69.4%)	26 (45.6%)	1	1
≥56	44 (30.6%)	31 (54.4%)	2.71 (1.44–5.08) **	1.50 (0.47–4.78)
Employment status				
Employed	89 (61.8%)	26 (45.6%)	0.52 (0.29–0.96) *	0.92 (0.34–2.49)
Unemployed	55 (38.2%)	31 (54.4%)	1	1
Remoteness				
Major cities of Australia	116 (80.6%)	35 (61.4%)	1	1
Regional and remote Australia	28 (19.4%)	22 (38.6%)	2.60 (1.33–5.11) **	1.11 (0.42–2.89)
Alcohol drinking				
No	49 (34.1%)	30 (52.6%)	2.15 (1.15–4.02)*	1.16 (0.45–2.98)
Yes	95 (65.9%)	27 (47.4%)	1	1
Level of social support				
Good social support	55 (38.2%)	43 (75.4%)	4.97 (2.49–9.91) **	3.28 (2.30–6.27)*
Poor social support	89 (61.8%)	14 (24.6%)	1	1
Level of stress				
No symptom of stress	76 (53.1%)	43 (75.4%)	2.70 (1.36–5.38) **	0.57 (0.17–1.90)
Symptom of stress	67 (46.9%)	14 (24.6%)	1	1
Level of anxiety				
No symptom of anxiety	64 (44.4%)	49 (86%)	7.66 (3.38–17.32) **	4.41 (3.64–14.68)*
Symptom of anxiety	80 (55.6%)	8 (14%)	1	1
Level of depression				
No symptom of depression	79 (54.9%)	49 (86%)	5.04 (2.23–11.39) **	1.80 (0.46–7.07)
Symptom of depression	65 (45.1%)	8 (14%)	1	1
Level of nicotine dependence				
Low dependence	98 (68.1%)	25 (43.9%)	1	1
Moderate to high dependence	46 (31.9%)	32 (56.1%)	2.73 (1.45–5.11) **	3.53 (1.40–8.95) **
Previous SCMs use				
Yes	83 (69.7%)	48 (90.6%	4.16(1.53–11.32) **	3.87 (1.11–13.44) *
No	36 (30.3)	5 (9.4%)	1	1
Type of SCM used				
NRT	108 (75%)	36 (63.2)		
Varenicline	22 (15.3%)	18 (31.5%)	2.45 (1.18–5.08) *	1.48 (0.53–4.13)
Bupropion	14 (9.7%)	3 (5.3%)	0.64 (0.17–2.36)	0.24 (0.39–1.49)
Ever forget to take the SCMs				
Yes	75 (52.1%)	19 (33.3%)	1	1
No	69 (47.9%)	38 (66.7%)	2.17 (1.15–4.12)*	1.67 (0.61–4.57)
Relapse				
Yes	99 (68.7%)	30 (52.6%)	1	1
No	45 (31.3%)	27 (47.4%)	1.98 (1.05–3.71) *	2.88 (1.21–6.88) *

* *p*-value <0.05; ** *p*-value <0.01; SCMs—smoking cessation medications; NRT—nicotine replacement therapy; COR—crude odds ratio; AOR—adjusted odds ratio.

## Data Availability

All relevant materials and data supporting the findings of this study are contained within the manuscript. However, if you need additional information, you can access the data from the corresponding author on responsible request.

## Abbreviations

FTND	Fagerstrom Test for Nicotine Dependence
DASS	Depression, Anxiety, and Stress Scale
NRT	Nicotine replacement therapy
OSSS	Oslo Social Support Scale
SCM	Smoking cessation medication
